# How to prepare chlorhexidine eye drops

**Published:** 2023-05-22

**Authors:** Abeer H A Mohamed-Ahmed, Christina A R Picken, Dan Kuguminkiriza

**Affiliations:** Research Fellow in Pharmacology and Clinical Trials Management: London School of Hygiene & Tropical Medicine, UK.; Research Fellow: School of Pharmacy, University College London, UK.; Pharmacist: Eye Drop Production Unit, Ruharo Eye Centre, Ruharu Mission Hospital, Mbarara, Uganda.

**Preparing chlorhexidine eye drops in a buffered acetate solution can help to improve patient comfort; here is how.**

**Figure 1:**
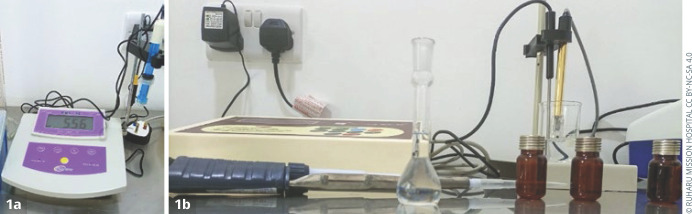
**a.** pH meter to measure the pH of buffer solutions and eye drops. **b.** Conductivity meter for water analysis.

Chlorhexidine eye drops can be used for the treatment of fungal keratitis as a second line therapy where natamycin eye drops are not available.^1^,^2^ Chlorhexidine eye drops typically used in the clinic are prepared by diluting a concentrated solution of chlorhexidine using sterile water without controlling for pH or tonicity^3^ and are reported to cause patient discomfort (stinging sensation). The pH and tonicity of chlorhexidine eye drops can be controlled by using acetate buffer (141.4 mM, pH 6.75) to improve patient tolerance/comfort.^3^ Controlling the pH of the chlorhexidine eye drops can also improve the stability of the drops: chlorhexidine eye drops prepared using acetate buffer (141.4 mM, pH 6.75) were shown to have stable pH (~6.75) and drug concentration at 40 degrees Celsius for 21 months.^3^

In this article, we share the protocol we used for the local preparation of acetate-buffered chlorhexidine eye drops at Ruharo Mission Hospital's eye drop production facility in Uganda.^3^ This protocol will produce 2,000 ml of buffered chlorhexidine eye drops, which is enough to fill 200 bottles containing 10 ml eye drop solution per bottle.

## Facilities/space

Ideally, eye drops should be prepared in a certified clean room equipped with an air control system complete with a filter that removes dust and other potential sources of contamination. A laminar flow cabinet can be used for local preparation of eye drops in the certified clean room or in a normal room with air conditioning. In the absence of a clean room/air control system and laminar flow cabinet, we recommend you take the following measures:

- Use a room that is dedicated for eye drop production only, with air conditioning and a double door entry system to avoid air disturbance, minimising contamination (airlock system).
- Less than 2 hours before each eye drop production session, clean the floors and surfaces of the production room (or the room and airlock) by wiping with distilled water and an antiseptic (such as Dettol or cetrimide 0.5% w/v), followed by spraying with 75% ethanol. Follow the same cleaning procedure after using the production room.
- Once surfaces are dry, spray production surfaces with 70% ethanol to further reduce the presence of microbes.

## What you will need

### Ingredients

- Chlorhexidine digluconate solution (20% w/v)
- Sodium acetate
- Acetic acid (20% v/v)
- Sodium hydroxide (10 M)
- Freshly distilled water

### Equipment

- Volumetric flasks (20 ml, 2000 ml) + stopper
- Measuring cylinders (10 ml and 1000 ml).
- Pipette (1 ml) and pipette tips
- Metal jug
- Filtration system (filter funnel, 5 µm filter membrane, filter clamp/support, vacuum pump, conical flask/bottle)
- Syringes (with 10 ml graduations)
- Amber glass eye drop 10 ml bottles
- Bottle lids – with or without dropper (Use HDPE or PP)
- Class II or electronic balance
- pH meter (Figure 1a)
- Conductivity meter (Figure 1b)
- Autoclave or steam bath
- Labels

### Method

- Wash hands before entering airlock room.
- Wear protective clothes (production gown, gloves, boots, hair net or head cap, mask and eye protection) in the airlock room.
- Ensure the production room and equipment are clean.
- Rinse all containers three times using distilled water, by filling them to the top and then discarding the water. Then sterilise using an autoclave or water bath.
- Wash and sterilise the empty eye drop bottles at 121°C for 15 minutes.
- Wash the filtration system by filling it with hot distilled water, flushing it using a vacuum pump, and discarding the water. Repeat this three times. Then backwash the filtering system three times by reversing the filtering head and filling the filtration system with hot distilled water using a vacuum pump.
- Collect 2,500 ml freshly distilled water.

### Prepare acetate buffer

- Dissolve sodium acetate (23.198 g) in 2,000 ml of freshly distilled water in a large mixing beaker. Then add 4.46 ml of acetic acid (20% v/v) to the sodium acetate solution and mix the solution.
- Using a portable pH meter, adjust the pH by dropwise addition of sodium hydroxide (10 M), adding approximately 1.938 ml to reach pH 6.75.

### Prepare chlorhexidine 0.2% (2,000 ml batches)

**Figure 2:**
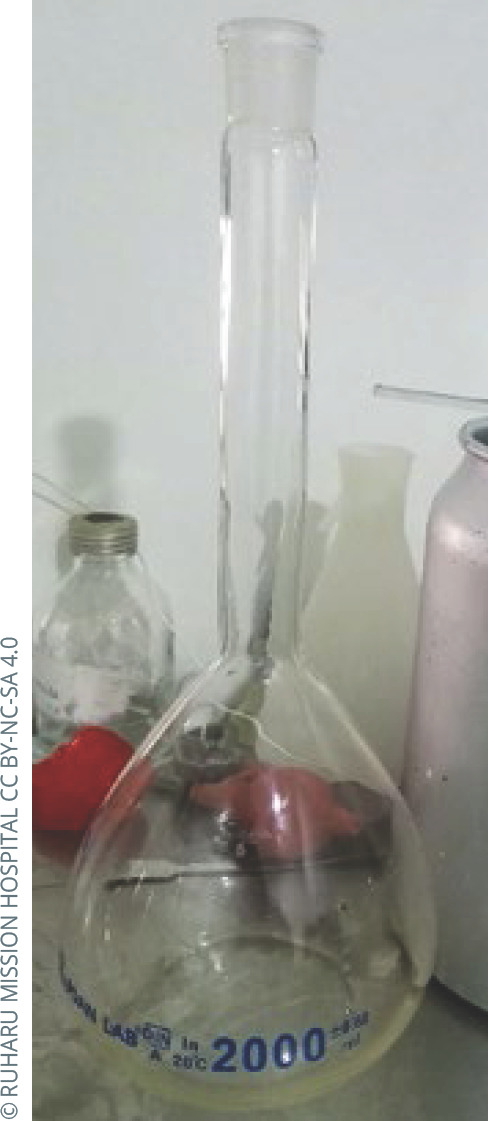
Volumetric flask (2,000 ml) for dilution of chlorhexidine 20% w/v to 0.2% w/v by addition of acetate buffer.

- Measure 20 ml of 20% chlorhexidine using 20 ml volumetric flask.
- Transfer 20 ml of chlorhexidine (20%) into 2,000 ml volumetric flask (Figure 2). Rinse the 20 ml flask three times with buffer and transfer the rinsed solution into the 2,000 ml volumetric flask (Figure 2).
- Fill the 2,000 ml volumetric flask containing the chlorhexidine solution to 2,000 ml mark with the acetate buffer.
- Place lid on flask, invert flask and shake to stir.
- Assemble filter membrane onto filter bed and secure with clamp/support. Pass solution through filter into rinsed conical flask/bottle using pump vacuum.
- Transfer filtrate into a jug or a bottle.

### Dispensing

**Figure 3:**
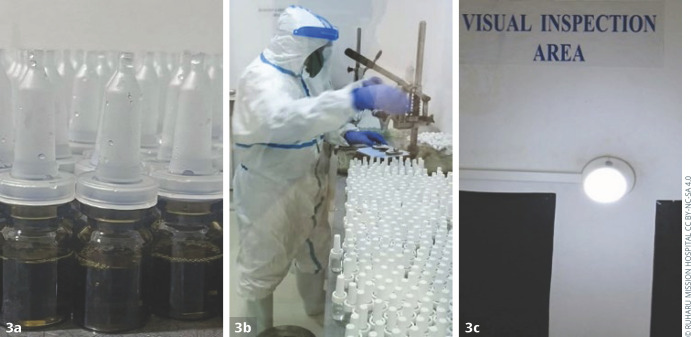
**a.** Amber glass bottles with PPE plastic droppers. **b.** Manual capping/sealing of bottles. **c.** Visual inspection area.

- Lay out the empty eye drop bottles on a clean surface.
- Dispense the eye drop solution using syringes or measuring cylinder or pressmatic pump dispenser in 10 ml portions into the amber glass eye drop bottles.
- Seal the bottles with tightly fitted lids (either polypropylene plastic dropper or HDPE cap) (Figure 3a) using a manual capping machine (Figure 3b) or by hand if a capping machine is not available.

### Sterilisation

- Sterilise the bottles using a water steam bath at 100°C (at atmospheric pressure) for 30 minutes. If an autoclave is available, sterilise by autoclaving at 121°C for 15 minutes.

### Visual inspection and quality control testing

- Do a visual inspection in bright light. Shake the eye drop bottle and observe the contents against white and black backgrounds to check for visible particles or debris (Figure 3C).
- Check lids cannot be tightened further.
- Check correct fill volume.
- Check for the presence of leaks by turning the filled eye drop bottles upside down.
- Send the first, middle, and last bottles of each batch for sterility testing and recall the batch immediately if any samples are positive for microorganisms.
- Check drug concentration, ideally using high performance liquid chromatography (HPLC). In case HPLC is not available, use a UV spectrophotometer to check the concentration of chlorhexidine in the eye drop solution.

### Labelling

Allow the sterilised bottles to stand for at least 12 hours to cool and dry before labelling.

The label should contain:

- Name and strength of the drug: chlorhexidine digluconate 0.2% w/v.
- Composition of the eye drops: pH buffered in acetate buffer, no added preservative.
- Storage conditions: store below or at 25°C and protect from light. Once opened, store at 4°C and use within 7 days.
- Shelf life of unopened sterile bottles: 24 months at 25°C.
